# Efficacy of TIMOLOL nasal spray as a treatment for epistaxis in hereditary hemorrhagic telangiectasia. A double-blind, randomized, placebo-controlled trial

**DOI:** 10.1038/s41598-019-48502-9

**Published:** 2019-08-19

**Authors:** Sophie Dupuis-Girod, Vincent Pitiot, Cyrille Bergerot, Anne-Emmanuelle Fargeton, Marjolaine Beaudoin, Evelyne Decullier, Valentine Bréant, Bettina Colombet, Pierre Philouze, Frédéric Faure, Jean-Charles Letievant

**Affiliations:** 1 Hospices Civils de Lyon, Hôpital Femme-Mère-Enfants, Service de Génétique et centre de référence pour la maladie de Rendu-Osler, Bron, F-69677 France; 2 Hospices Civils de Lyon, Hôpital E. Herriot, Service d’ORL, Lyon, F-69437 France; 3 Hospices Civils de Lyon, Hôpital de la Croix Rousse, Service d’ORL, Lyon, F-69317 France; 4 Hospices Civils de Lyon, Hôpital Louis Pradel, Service de cardiologie, Lyon, F-69677 France; 5 Hospices Civils de Lyon, pôle Santé Publique, Lyon, F-69003 France; 6 Université Lyon 1, F-69008 Lyon, France; 7 Hospices Civils de Lyon, Pharmacie, Hôpital Louis Pradel, Bron, F-69677 France

**Keywords:** Cardiovascular diseases, Randomized controlled trials

## Abstract

Hereditary hemorrhagic telangiectasia is a rare vascular genetic disease. Epistaxis is the most frequent and disabling manifestation, and timolol appears to be a new therapeutic option as non-selective beta-blockers have *in vitro* and *in vivo* anti-angiogenic properties. Our main objective was to evaluate the efficacy of TIMOLOL nasal spray as a treatment for epistaxis in hereditary hemorrhagic telangiectasia. This study is a single-center, randomized, phase 2, double-blind placebo-controlled study with an allocation ratio of 1:1. It was proposed to patients with hereditary hemorrhagic telangiectasia monitored at the French Reference Center, and we included patients aged over 18 years, diagnosed with hereditary hemorrhagic telangiectasia and epistaxis. The treatment was self-administered by the patient with a posology of one spray (50 µL) of timolol 0.5% or placebo in each nostril twice a day for 28 consecutive days. The primary efficacy endpoint was mean monthly epistaxis duration, assessed by monitoring epistaxis grids. A total of 58 patients were randomized and treated. The baseline characteristics were similar in the 2 groups. Mean monthly epistaxis duration measured at 3 months was not significantly different in the 26 patients receiving the drug in comparison with the placebo group (p = 0.54). Toxicity was low and no severe adverse events were reported. One limitation is that we included all HHT patients with nosebleeds and did not take into account history of nasal surgery or nasal crusts. Timolol, administered by nasal spray at a dose of 0.25 mg in each nostril twice a day for 28 consecutive days, did not improve epistaxis in patients with hereditary hemorrhagic telangiectasia at 4 months after the beginning of the treatment.

## Introduction

Hereditary hemorrhagic telangiectasia (HHT) is a genetic vascular disorder (HHT; Online Mendelian Inheritance in Man® #187300) characterized by recurrent epistaxis, telangiectasia and visceral arteriovenous malformations (AVM) affecting lungs, liver, gastrointestinal tract and brain. Diagnosis is based on the Curaçao criteria^1^ and is considered definite if at least three of four criteria are fulfilled (epistaxis, telangiectasia, family history and visceral lesions).

The most frequent expression of HHT is the occurrence of repeated severe and disabling epistaxis^2^ which can cause chronic anemia, and can require iron supplementation and multiple blood transfusions. Management of this major symptom is not well-established and often demands local treatments or medication whose efficacy is not sufficiently documented^3,4^. There is no current surgical treatment that makes it possible to cure the nosebleeds definitively. Furthermore, the repetition of aggressive surgical treatments is often the source of significant iatrogenic conditions, including the perforation of the nasal septum, resulting in a worsening of the nosebleeds.

HHT is related to an imbalanced state between anti-angiogenic factors and pro-angiogenic factors^5^ secondary to mutations in three genes, *ENG* (encoding endoglin)^6^, *ACRLV1* (encoding activin receptor-like kinase 1)^7^ and *MADH4 (*encoding SMAD4). Of the drugs with an anti-angiogenic effect^8^, propranolol, a non-selective beta-adrenergic receptor blocker was a good candidate, showing antiproliferative and apoptotic effects on human umbilical vein endothelial cells^9^, and reducing vascular endothelial growth factor (VEGF)^10^, thus inhibiting angiogenesis. Furthermore, propranolol decreased *in vitro* endoglin, ACVRL1 mRNA and protein levels^11^. Propranolol anti-angiogenic properties were demonstrated in 2008 in the treatment of infantile hemangioma^12^ and in HHT, with one case report indicating that intranasal timolol (0.5% ophthalmic solution) reduced the frequency and severity of epistaxis in one HHT patient, after 3–4 days of treatment^13^. Furthermore, timolol solution was commercialized and easy to use.

On the basis of these encouraging results, and based on previous study results using anti-angiogenic treatments in HHT like bevacizumab and thalidomide^8^, we planned a phase 2 study to evaluate, over a 3 month-period after the end of the treatment, the efficacy on the duration of nosebleeds at a dose of 50 µL timolol (total of 1 mg/d) *vs* placebo administered in each nostril twice a day for 28 consecutive days in patients with HHT complicated by nosebleeds (main outcome).

## Patients and Methods

### Study overview

The study was prospective, placebo-controlled, comparative and single-center. This study was approved by the local research ethics committee (CPP) and by the French Medical Products Agency (ANSM) in March 2015. Written informed consent was obtained from all patients in accordance with national regulations. The trial was conducted in accordance with the principles of the Declaration of Helsinki^14^ and Good Clinical Practice guidelines. All the authors were involved in the design or conduct of the study, as well as the preparation of the manuscript and the decision to submit it for publication. This trial was registered with the ClinicalTrials.gov Identifier #NCT02484716 (TEMPO study, date of registration 30/06/2015).

### Patient population

This study enrolled patients over the age of 18 years with clinically confirmed HHT, suffering from epistaxis (more than 60 minutes as a total over a three month-period assessed using specific grids filled in by the patients), and who had not undergone nasal surgery in the 3 months prior to inclusion.

We did not include women who were pregnant or those likely to become so during the study, patients with bronchial asthma or chronic pulmonary disease, patients with low blood pressure, or cardiac failure, or bradycardia, or other cardiac contraindications to beta blockers, or patients with pheochromocytoma or severe peripheral circulatory disturbances. We did not include patients with an ongoing treatment comprising calcium antagonists or antiarrhythmics or clonidine, lidocaine, beta-blocker treatment or floctafenine, sultopride or amiodarone treatments, or those with known hypersensitivity to the active ingredients or one of the excipients, or patients who had incompletely filled in the nosebleed grids in the 3 months preceding the treatment. Potentially eligible patients were identified in the HHT network and informed during a standard ear, nose and throat (ENT) consultation or in the Reference Center or Skill Centers.

### Study design

This is a prospective, double-blind, phase IIb study, with a randomization scheme comprising an equal active/placebo ratio (1:1).

Randomization was performed by a statistical department for the allocation of verum or placebo. The randomization list was established using SAS software version 9.4 (SAS Institute Inc., Cary, NC, USA).

All clinical and biological data were collected during consultations at the HHT Reference Center (Lyon) on electronical secured software dedicated to the study.

The treatment was self-administered by the patient with a posology of one spray (50 µL) in each nostril twice a day for 28 consecutive days. The product, a marketed ophthalmic solution with timolol at 0.5% or placebo, was packaged by a pharmaceutical department in a calibrated nasal spray bottle that delivered 50 µL per nebulization. The spray was given to the patient on the day of inclusion. The placebo used was 0.9% sodium chloride.

At inclusion, an electrocardiogram was performed if it had not been recorded in the previous year and a cardiology consultation verified the absence of contraindications to beta-blocker treatment. Patients had 6-month follow-up with visits and a blood sample (hemoglobinemia, ferritinemia) at the end of the treatment (day 28) and 3 and 6 months after, including a physical examination and/or ENT consultation.

### Study endpoints

The primary efficacy endpoint was the improvement rate for epistaxis. An improvement was defined as a decrease of at least 30% in the mean monthly epistaxis duration of nosebleeds during the three-month period immediately after treatment, compared to the three-month period prior to inclusion.

Secondary outcomes were average monthly frequency (number/month) of nosebleeds, quality of life, evaluated with the SF36 quality of life questionnaire, number of red blood cell transfusions, biological efficacy criteria (hemoglobin and serum ferritin) and safety. All were evaluated before treatment and at 3 and 6 months after the end of the treatment.

### Sample size calculation and statistical analysis

Treatment with the timolol nasal spray was judged effective if at least half of the patients improved. We hypothesized that 15% of patients would improve in the placebo group (versus 50% in the treatment group). It was necessary to include 26 patients in each group, with 52 patients overall to reach 80% power with a 5% alpha according to a bilateral hypothesis. Taking into account early withdrawal and patients who may be lost to follow-up, we had to include 29 patients in each group, that is to say, a total of 58 patients.

The primary analysis was performed in the intention-to-treat (ITT) population, which included all the patients who had undergone randomization and were confirmed to have started the study. Patients were analyzed in their randomization group regardless of the treatment received. Per protocol (PP) population was defined as all patients without major deviation.

The initial characteristics of the patients were summarized by means of descriptive statistics (number, average, standard deviation, median, minimum and maximum for the quantitative variables and numbers and percentages for the qualitative variables).

#### Analysis of the main judgment criterion

First, total duration was computed for each patient by totaling all epistaxis durations for the period considered. This was then divided by the number of values available for the period and multiplied by 28 days (i.e. 4 weeks) to obtain a monthly mean. This computation made it possible to overcome missing data and to harmonize different numbers of days per month.

The percentage of patients experiencing improvement was computed in each group and was compared using a Chi² test (or a Fischer’s test if the conditions of the Chi² test could not apply). This analysis was performed on the ITT population and confirmed on the per protocol population. As a complement, the details of the durations used for the main criterion will be presented and compared with a Student t-test (or Mann-Whitney test in case of non-normality).

Analysis of the secondary judgment criteria: evolution in clinical, paraclinical and quality of life parameters were compared between groups using Student’s t-tests (or Mann-Whitney’s tests in case of non-normality).

The percentages of occurrence of AEs and SAEs was computed in each group and compared by means of a Chi² test (or a Fischer’s test if the conditions of the Chi² test could not apply).

All analyses were performed using SAS software version 9.4 (SAS Institute Inc., Cary, NC, USA).

The trial statisticians had full access to all the data and assumed responsibility for the integrity of the data, the completeness and accuracy of the data and analysis, and the coherence of the trial with the protocol.

### Safety

Safety was evaluated at each visit by a physical examination (monitoring of blood pressure, clinical ear, nose and throat examination to check the nasal septum and other side effects on the nasal mucosa), laboratory testing, and assessment for adverse events. Adverse events were classified as non-related, or related to the treatment. Monitoring the safety of administration of the product, motivated by the iatrogenic risks, justified the setting up of a specific independent monitoring and safety committee. The committee met in particular in the case of the occurrence of serious adverse events. It was composed of a specialist in the disease; a cardiologist, and a statistician specializing in the methodology of clinical trials but not involved in the study.

## Results

### Trial population

**Figure 1:**
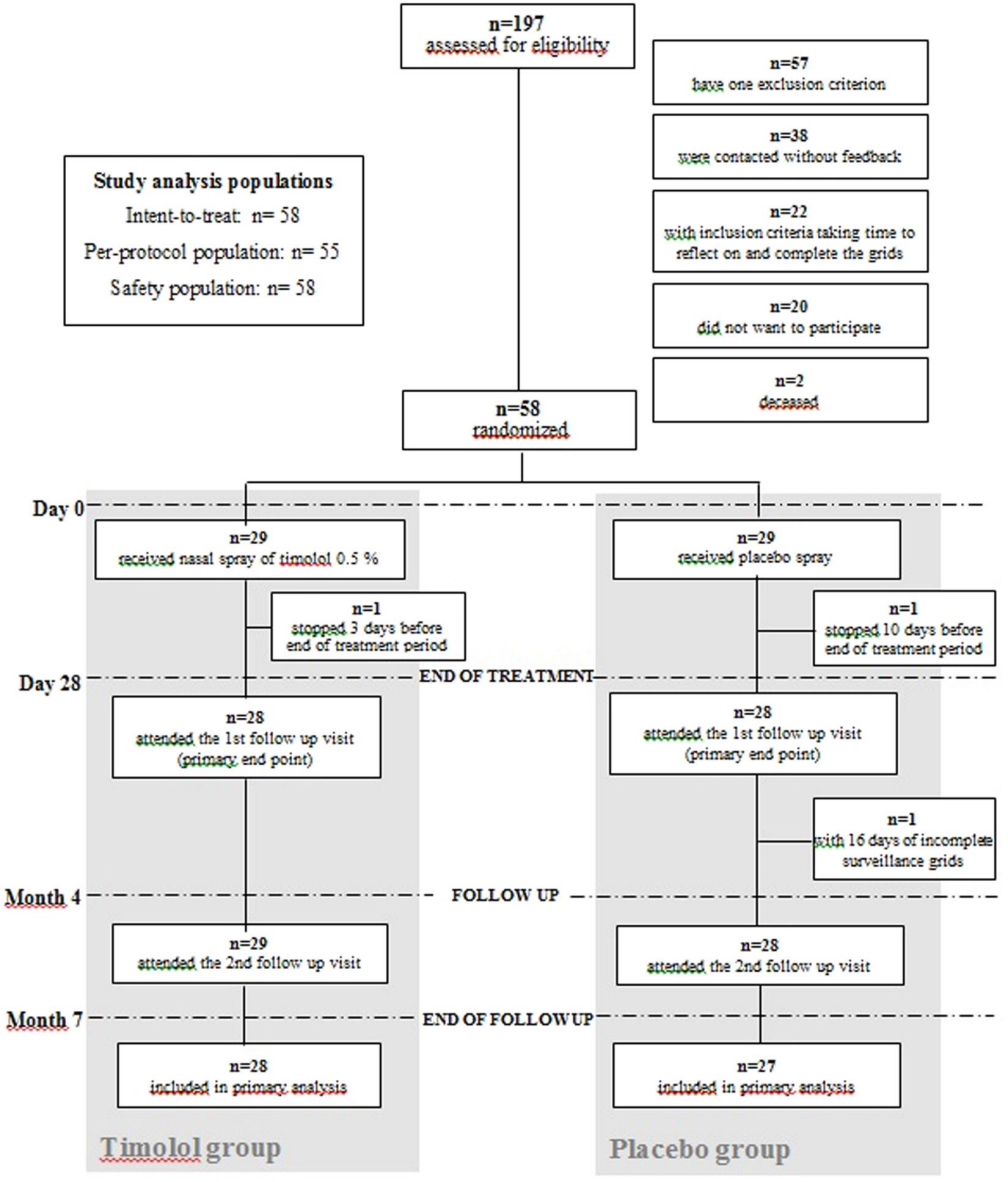
Enrollment and outcomes.

Of the 197 patients assessed for eligibility, 58 were randomized between June 2015 and June 2017 (Fig. 1). The baseline characteristics are summarized in Table 1 and were similar in both groups. Septal perforation frequency was similar in both groups and no changes during treatment were observed. Of them, 29 received timolol and 29 received placebo. All were included in the primary analysis.

**Table 1:** Patients’ characteristics before treatment.

Variable	Modality	Placebo group	Timolol Group	p-value
n		29	29	—
Age (years)	Mean (SD)	56.5 (7.9)	55.7 (10.8)	0.76
	Median (Min - Max)	56.8 (41.8–77.5)	53.5 (31.2–77.4)	
Females (%)	n (%)	17 (58.6)	14 (48.3)	0.43
Nasal surgery	n (%)	23 (79.3)	22 (75.9)	0.75
Nasal septum perforation	n (%)	7 (25.0)	8 (27.6)	0.82
Nasal obstruction	n (%)	23 (79.3)	23 (79.3)	1.00
Blood transfusions in the last 3 months	n (%)	5 (17.8)	4 (13.8)	1.00
Hemoglobinemia	Mean ± SD	117.62 (24.9)	129.21 (22.7)	0.09
(g/L)	Median (Min − Max)	119 (79–160)	134 (91–161)	
Ferritinemia (µg/l)	Mean ± SD	35.62 (39.1)	42.76 (63.7)	0.61
	Median (Min − Max)	23 (6–189)	23 (7–350)	
Systolic blood pressure (mmHg)	Mean ± SD Median (Min − Max)	128.2 (18.5) 130 (91–185)	130.0 (12.5) 128 (109–159)	0.68
Diastolic blood pressure (mmHg)	Mean ± SD Median (Min − Max)	80.4 (12.1) 78 (61–116)	78.7 (10.4) 77 (61–97)	0.57
Mutated gene	n (%)			1.00
ALK1		18 (62.1)	19 (65.5)	
ENG		9 (31.0)	9 (31.0)	
SMAD4		1 (3.4)	0 (0.0)	
On going		0 (0.0)	1 (3.4)	
Unknown		1 (3.4)	0 (0.0)	

All patients, except 2, completed 4 weeks of treatment and received the treatment as indicated and all were included in the primary analysis but excluded from the PP population. One patient in the placebo group stopped the treatment 10 days before the end for headache and diffuse pains, and one patient in the timolol group stopped two days before the end of the treatment because of bradycardia. One more patient of the placebo group was excluded from the PP population due to missing data in the epistaxis grid. The PP population therefore consisted of 55 patients.

### Response to treatment

#### Primary endpoint at intermediate analysis

**Figure 2:**
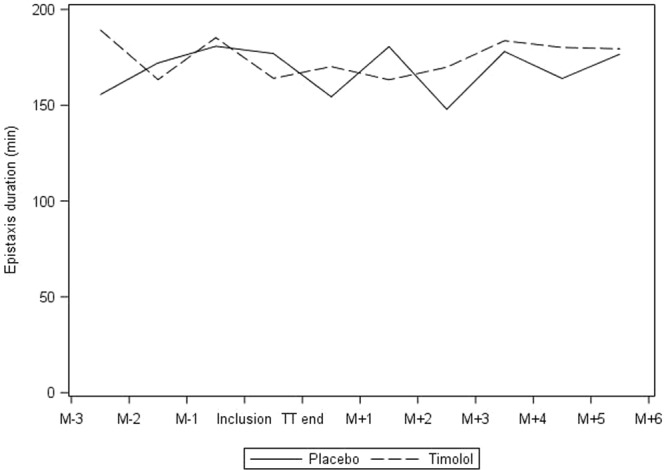
Mean monthly epistaxis duration before and after treatment.

Results of the analysis are summarized in Table 2 and Fig. 2. There was no significant difference in the proportion of patients who experienced a reduction of over 30% in mean epistaxis duration after treatment between the groups: 6 out of 29 (20.7%) patients had improved in the placebo group *versus* 8 out of 29 (27.6%) in the timolol group (p = 0.54). This result was also confirmed on the per protocol population.

**Table 2:** Details of durations used for the main criterion in each group.

	Variable	Overall	Placebo Group	Timolol Group	P-value
**Total epistaxis duration before treatment**					
M − 3	n	58	29	29	0.47
	median (min − max)	108.5 (0–825)	83 (0–545)	143 (22.58–825)	
	Mean (SD)	172.47 (177.43)	155.6 (150.97)	189.33 (201.76)	
M − 2	n	58	29	29	0.99
	median (min − max)	122.5 (15–600)	118 (15–600)	129 (22.15–561)	
	Mean (SD)	167.74 (139.12)	172.1 (151.46)	163.38 (128.13)	
M − 1	n	58	29	29	0.97
	median (min − max)	155.5 (0–580)	128 (0–520)	163 (12.5–580)	
	Mean (SD)	183.04 (145.42)	180.79 (146.39)	185.29 (146.99)	
**Total epistaxis duration after treatment (3 months)**					
M + 1	n	58	29	29	0.62
	median (min − max)	125 (11–612)	100 (14–505)	143 (11–612)	
	Mean (SD)	162.3 (137.13)	154.42 (136.36)	170.18 (139.84)	
M + 2	n	58	29	29	0.54
	median (min − max)	94 (6–605)	95 (12–513)	93 (6–605)	
	Mean (SD)	171.95 (162.32)	180.61 (160.99)	163.29 (166.02)	
M + 3	n	58	29	29	0.96
	median (min − max)	109.5 (7–599)	97 (7–475)	126 (10.2–599)	
	Mean (SD)	158.87 (146.98)	147.89 (126.31)	169.85 (166.66)	
**Mean epistaxis duration at 3 months**					
Before treatment	n	58	29	29	0.78
	median (min − max)	136.5 (19.08–642.33)	132 (24–555)	141.67 (19.08–642.33)	
	Mean (SD)	174.57 (144.72)	169.5 (143.08)	179.65 (148.71)	
After treatment	n	58	29	29	0.98
	median (min − max)	123.17 (12.4–583.67)	101.67 (14–491.67)	125.33 (12.4–583.67)	
	Mean (SD)	164.92 (141.94)	162.06 (134.45)	167.78 (151.39)	
Difference Before - After	n	58	29	29	0.81
	median (min − max)	−18.17 (−230.33–134.33)	−16.33 (−230.33–134.33)	−20 (−177.33–125)	
	Mean (SD)	−9.65 (68.61)	−7.44 (71.23)	−11.87 (67.08)

#### Secondary outcomes

Three and 6 months after the end of the treatment, no significant difference was observed between the groups in terms of number of epistaxes. Mean number of epistaxes at 3 consecutive months prior to inclusion and 3 months after the end of the treatment was 90.9 (SD 72.9) vs 82.4 (SD 75.4) in the placebo group and 94.1 (SD 65.3) vs 86.0 (SD 62.9) in the timolol group. The evolution was no different between the 2 groups (p = 0.79).

The SF-36 questionnaire revealed no significant evolution in any dimensions of quality of life after treatment (bodily pain (p = 0.12), general health (p = 0.88), mental health (p = 0.12), physical functioning (p = 0.69), social functioning (p = 0.73) and vitality (p = 0.10) (Figure in Supplemental Material).

The evolution in the number of blood transfusions before and after treatment was not significantly different either (p = 0.72), nor were the biological criteria (ferritinemia and hemoglobinemia) (p = 0.21 and 0.66 respectively). Mean hemoglobin level (g/L) at inclusion, month 3 and month 6 after treatment were 117.6 (24.9), 121.9 (SD 23.0) and 119.9 (SD 28.1) in the placebo group (p = 0.09, 0.17, 0.25 respectively) and 129.2 (SD 22.7), 129.3 (SD 25.8), 128.0 (SD 24.6) in the timolol group.

#### Given these negative results, we compared epistaxis duration before and during treatment

There was no significant difference. Mean epistaxis duration the month before treatment and during treatment was respectively 179.6 min (SD = 148.7) and 164.1 min (SD = 149.6) in the timolol group and 169.5 min (SD = 143.1) and 177 min (SD = 140.1) in the placebo group (p = 0.38).

#### Adverse effects

Three grade 2 adverse events (AE) certainly related to the treatment were recorded in 2 patients treated in the timolol group (bradycardia (n = 2) and low blood pressure (n = 1)) (Table 3). The two cases of bradycardia were considered as SAE, the treatment was stopped in one case and the bradycardia resolved spontaneously. Interestingly, the response to the treatment was good in one case and excellent in the second case) (Figure in Supplemental Material). No case of bradycardia was observed in the placebo group. Other related (probably or possibly) adverse events reported were all emergent-adverse events and included asthenia, headache, malaise, dizziness, insomnia, diffuse pain, dyspnea and nasal obstruction. No deaths were reported.

**Table 3:** Safety.

Adverse events		Overall n	Placebo group n (%)	Timolol group n (%)	P-value
Number of patients with at least one AE	n	31	15	16	0.88
Total number of adverse events	n	66	29	37	
Grade 1	n	12	6 (20.7)	6 (16.2)	
Grade 2	n	49	18 (62.1)	31 (83.8)	
Grade 3	n	5	5 (17.2)	0 (0)	

## Discussion

This was the first phase 2, prospective, randomized, controlled trial comparing timolol nasal spray to placebo in a double-blind setting in HHT patients. Contrary to our expectations, timolol did not reduce the duration of epistaxis in HHT patients compared to placebo. None of the secondary outcomes improved either. As observed in other studies in HHT^15,16^, epistaxis duration is highly variable from one patient to another and in a same patient over time, thus good responses to a treatment in case reports^13^ or small series without a placebo group^17^ need to be interpreted with caution.

We decided to use the marketed ophthalmic solution with timolol at 0.5% in a calibrated nasal spray bottle that delivered 50 µL per nebulization, without any changes of galenic formulation. However, we can hypothesized that the formulation could be improved and that a gel stays longer on patients’ nasal mucosa and more of the active substance can be absorbed. Indeed, preliminary results published by Mei-Zahav M *et al*.^18^ on 6 patients with HHT and treated with 0.5 cm^3^ of 1.5% propranolol gel, applied to each nostril twice daily for at least 12 weeks were encouraging. The gel was supplied by the patient with a plastic funnel-shaped guide attached to a syringe to avoid trauma to the nasal mucosa.

In our study, the 4-week treatment duration may not have been long enough for the full effect of the timolol to be expressed. However, in the case reported^13^, improvement was observed within 3 to 4 days, with a significant reduction in the frequency and severity of the epistaxes. The month after the initiation of treatment, nosebleed frequency had decreased to an average of 1 to 2 per week. The aim of our study was to assess whether these encouraging findings were a true effect of the treatment by including a placebo arm, which addresses the main limitation of the previous case. Contrary to the previous findings, our study shows that a 4-week treatment with timolol was not effective in the treatment of epistaxis in HHT. The question as to whether a longer duration of treatment might have been necessary remains formally unanswered. However, based on the disappointing results observed in the subset of patients undergoing the 4-week treatment with timolol, it is doubtful that longer treatment durations would prove to be effective.

Tolerance of intranasal timolol was good after a 4-week nasal spray administration in the present study. Unsurprisingly, bradycardia was observed in 2 cases (0.5%). No pharmakokinetics study was performed in this study. However, when administered intraocularly, on skin, as a treatment for infantile hemangiomas, or on nasal mucosa, timolol is known to be systematically absorbed with possible cardiovascular effects^19–21^. Timolol is metabolized by cytochrome P450 (CYP) 2D6 in the liver with a minor contribution of CYP2C19. Bradycardia is related to the CYP2D6 genotype^19,21^ and has been observed after local administration with beta-blockade even at low-plasma concentrations of timolol (1.0 ng/mL)^20^. Differences in enzyme activities lead to four major phenotypes: poor (PM), intermediate (IM), extensive (EM) and ultrarapid (UM) metabolizers^22^. Furthermore, the nasal mucosa is known to be an efficient route for drug delivery, and the significant telangiectasias associated with HHT may enhance the systemic uptake of drugs^23,24^. This risk was taken into consideration during patient follow-up with regular measurement of cardiac rate and blood pressure, and a systematic ECG was performed before beginning the treatment. Furthermore, it could be planned in further studies to evaluate the relationship between the magnitude of heart rate or blood pressure reduction and epistaxis duration.

Our trial had several limitations. First, even though HHT patients in France are used to completing epistaxis grids and detailing durations of epistaxis in HHT, the procedure is not precise. In other countries, The Epistaxis Severity Score (ESS) is used, taking into account frequency, duration and blood transfusion, but again is not accurate. For this reason, we decided to use the same tool (epistaxis grid), as in previous studies published on epistaxis in HHT^25–27^. Secondly, we included all HHT patients with nosebleeds and did not take into account history of nasal surgery or nasal crusts (which may change mucosa drug absorption), however almost all patients had had different types of surgery before receiving the nasal spray, as well as mucosal lesions, and it is difficult to evaluate possible absorption before treatment. Finally, we used SF36 questionnaires to measure quality of life in HHT patients because it has already been evaluated in HHT^28–30^, but it is not a specific tool for HHT disease.

In conclusion, timolol given by nasal spray for 4 weeks did not improve epistaxis duration in HHT patients. The treatment was safe in all cases.

## Supplementary information

Supplemental material

## Data Availability

The datasets used and/or analysed during the current study are available from the corresponding author on reasonable request.
