# Supplementary material for: Efficacy of TIMOLOL nasal spray as a treatment for epistaxis in hereditary hemorrhagic telangiectasia. A double-blind, randomized, placebo-controlled trial
Source: Sci Rep. 2019 Aug 19;9:11986. doi: 10.1038/s41598-019-48502-9 (PMC6700077; doi:10.1038/s41598-019-48502-9)

Supplemental material

Efficacy of TIMOLOL nasal spray as a treatment for epistaxis in hereditary hemorrhagic telangiectasia. A double-blind, randomized, placebo-controlled trial

Sophie Dupuis-Girod[1], MD, PhD, Vincent PITIOT[2], MD, Cyrille BERGEROT[4], MD, Anne-Emmanuelle Fargeton[1], MsC, Marjolaine Beaudoin[1], MsC, Evelyne Decullier[5,6], PhD, Valentine Bréant[7], Pharm D, Bettina Colombet[7], Pharm D, Pierre Philouze[3], MD, Frédéric Faure[2], MD, PhD and Jean-Charles Letievant[2], MD.

1. Hospices Civils de Lyon, Hôpital Femme-Mère-Enfants, Service de Génétique et centre de référence de la maladie de Rendu-Osler, Bron F-69677,
2. Hospices Civils de Lyon, Hôpital E. Herriot, Service d'ORL, Lyon, F-69437
3. Hospices Civils de Lyon, Hôpital de la Croix Rousse, Service d'ORL, Lyon F-69317
4. Hospices Civils de Lyon, Hôpital Louis Pradel, Service de cardiologie, Lyon F-69677
5. Hospices Civils de Lyon, pôle Santé Publique, Lyon, F-69003
6. Université Lyon 1, F-69008
7. Hospices Civils de Lyon, Pharmacie, Hôpital Louis Pradel, Bron, F-69677

Supplemental material

Figure 3: SF36 score before and after treatment in the placebo (A) and timolol (B) groups.

A

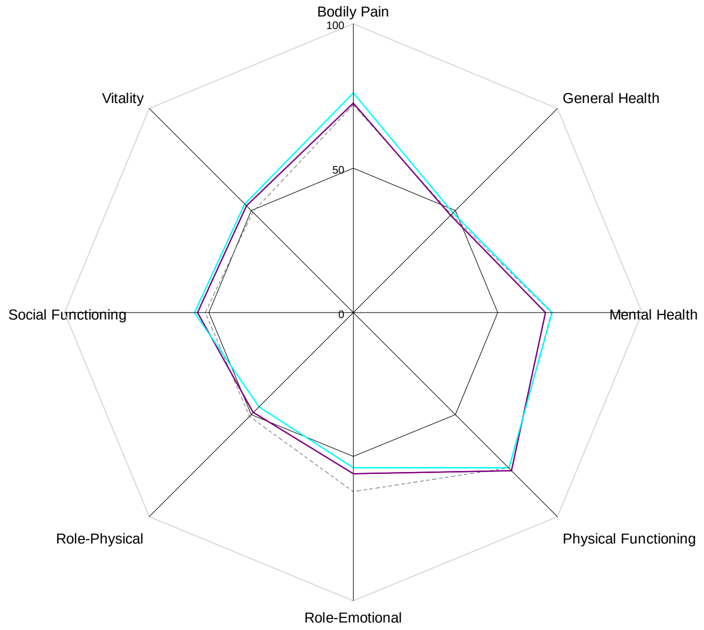

B

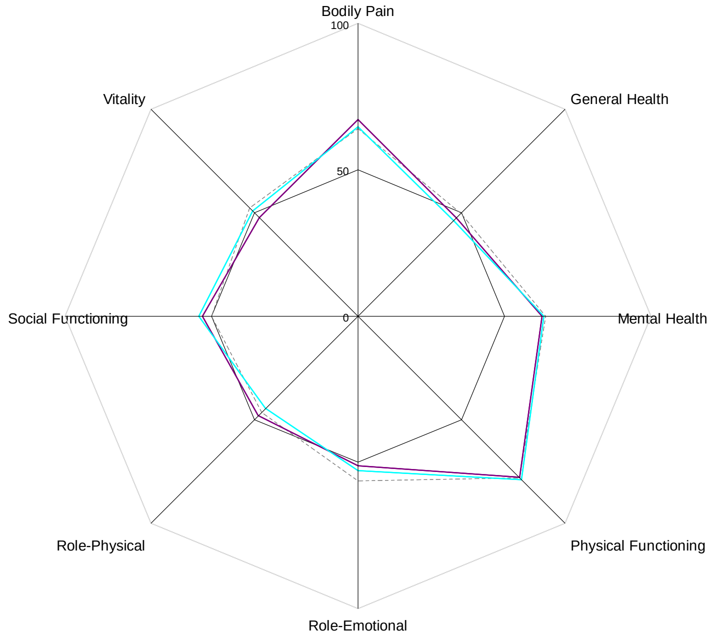

Legends: - - - **SF36 at inclusion**

SF36 at 4 months after inclusion

SF36 at 7 months after inclusion

Supplemental material

Figure 4: Epistaxis duration and cardiac frequency in the 2 patients who experienced bradycardia during treatment

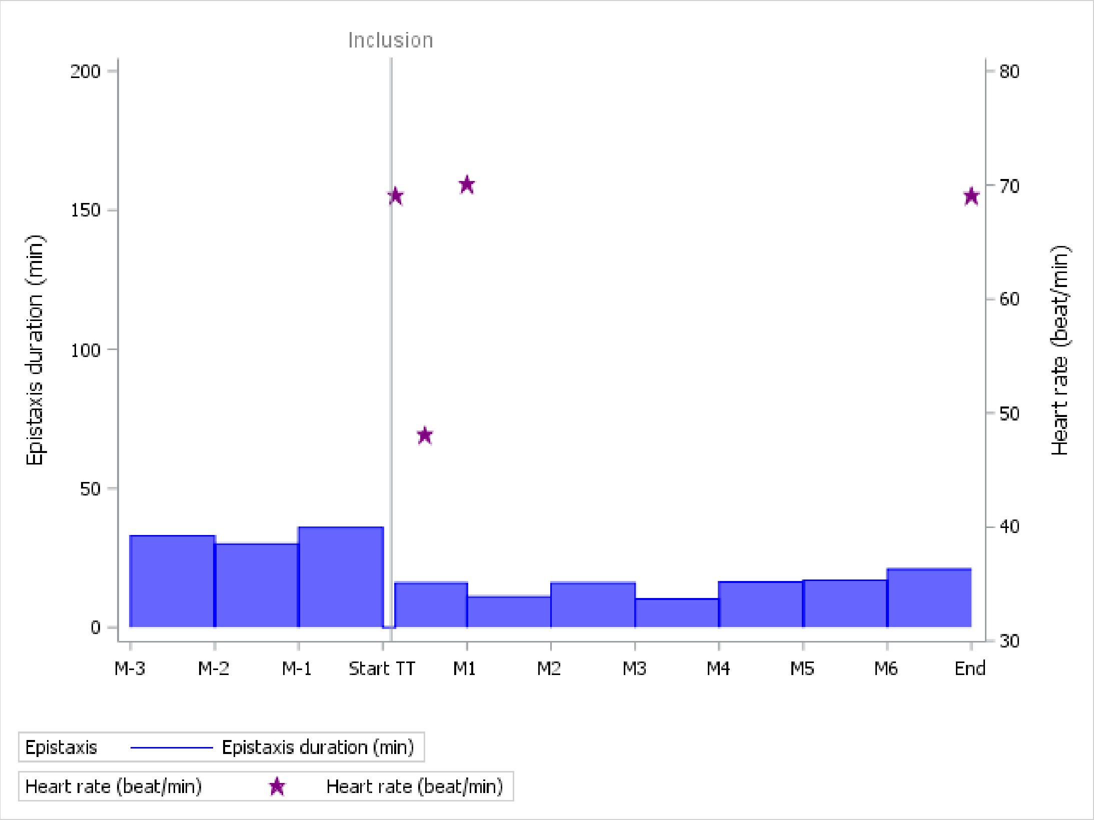

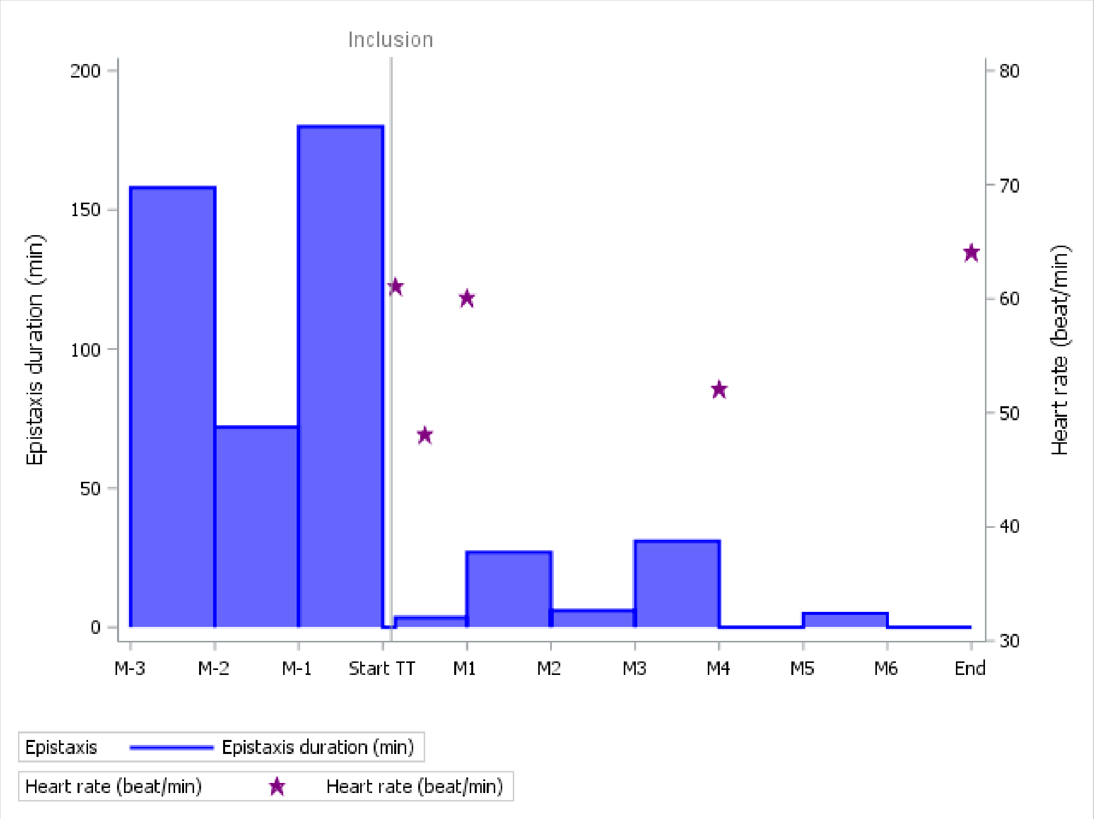

Supplement: Supplementary file 1 — Supplemental material [file 41598_2019_48502_MOESM1_ESM.pdf]
